# Transforming Growth Factor-β Induces Transcription Factors MafK and Bach1 to Suppress Expression of the Heme Oxygenase-1 Gene*[Fn FN2]

**DOI:** 10.1074/jbc.M113.450478

**Published:** 2013-06-04

**Authors:** Yukari Okita, Atsushi Kamoshida, Hiroyuki Suzuki, Ken Itoh, Hozumi Motohashi, Kazuhiko Igarashi, Masayuki Yamamoto, Tomohiro Ogami, Daizo Koinuma, Mitsuyasu Kato

**Affiliations:** From the ‡ Department of Experimental Pathology, Graduate School of Comprehensive Human Sciences and Faculty of Medicine, University of Tsukuba, 1-1-1 Tennodai, Tsukuba 305-8575, Japan,; § Center for Tsukuba Advanced Research Alliance, University of Tsukuba, 1-1-1 Tennodai, Tsukuba 305-8577, Japan,; ¶ Department of Stress Response Science, Hirosaki University Graduate School of Medicine, Hirosaki 036-8562, Japan,; ‖ Center for Radioisotope Sciences and; ** Departments of Biochemistry and; ‡‡ Medical Biochemistry, Tohoku University Graduate School of Medicine, 2-1 Seiryo-machi, Aoba-ku, Sendai 980-8575, Japan, and; §§ Department of Molecular Pathology, Graduate School of Medicine, University of Tokyo, Tokyo 113-0033, Japan

**Keywords:** Cancer, Heme Oxygenase, Nrf2, Oxidative Stress, Transforming Growth Factor β (TGFβ), Cancer Prevention, Transcriptional Regulation

## Abstract

Transforming growth factor-β (TGF-β) has multiple functions in embryogenesis, adult homeostasis, tissue repair, and development of cancer. Here, we report that TGF-β suppresses the transcriptional activation of the heme oxygenase-1 (*HO-1*) gene, which is implicated in protection against oxidative injury and lung carcinogenesis. *HO-1* is a target of the oxidative stress-responsive transcription factor Nrf2. TGF-β did not affect the stabilization or nuclear accumulation of Nrf2 after stimulation with electrophiles. Instead, TGF-β induced expression of transcription factors MafK and Bach1. Enhanced expression of either MafK or Bach1 was enough to suppress the electrophile-inducible expression of *HO-1* even in the presence of accumulated Nrf2 in the nucleus. Knockdown of MafK and Bach1 by siRNA abolished TGF-β-dependent suppression of *HO-1*. Furthermore, chromatin immunoprecipitation assays revealed that Nrf2 substitutes for Bach1 at the antioxidant response elements (E1 and E2), which are responsible for the induction of *HO-1* in response to oxidative stress. On the other hand, pretreatment with TGF-β suppressed binding of Nrf2 to both E1 and E2 but marginally increased the binding of MafK to E2 together with Smads. As TGF-β is activated after tissue injury and in the process of cancer development, these findings suggest a novel mechanism by which damaged tissue becomes vulnerable to oxidative stress and xenobiotics.

## Teaser

**Background:** TGF-β suppresses early carcinogenesis but accelerates malignant progression by activating invasion and metastasis. *HO-1* is induced in response to oxidative stress and protects cells from oxidative injury.

**Results:** TGF-β suppresses *t*BHQ-inducible expression of *HO-1* through induction of MafK and Bach1.

**Conclusion:** TGF-β suppresses a protective response to electrophiles.

**Significance:** This work provides the first evidence that TGF-β affects electrophilic responses.

## Introduction

Transforming growth factor-β (TGF-β) regulates multiple biological functions such as cell proliferation, differentiation, apoptosis, and morphogenesis (1, 2). Upon ligand binding, type II serine/threonine kinase receptors activate type I receptors, and activated type I receptors phosphorylate Smad proteins. Phosphorylated receptor-regulated Smads (R-Smads; Smad2 and Smad3) form heteromeric complexes with common partner Smad (Co-Smad; Smad4) and accumulate in the nucleus. Activated Smad complexes regulate expression of target genes by binding to specific DNA sequences together with various cobinding transcription factors and recruiting coactivators or corepressors (3). Differential expression of cobinding transcription factors contributes to the cell type- and context-dependent cellular responses to TGF-β.

TGF-β is a potent inhibitor of epithelial cell proliferation; therefore, it acts as a tumor suppressor in the early stages of carcinogenesis. On the other hand, cancer cells develop resistance to TGF-β-inducible growth inhibition in the advanced stages of carcinogenesis. At these later stages, TGF-β promotes epithelial-mesenchymal transition, invasion, and metastasis in certain types of cancer cells without TGF-β receptor abnormalities (4). Therefore, TGF-β is thought to act as a double-edged sword in cancer development (5). However, the multifunctional effects of TGF-β on cancer initiation and progression have not been fully elucidated.

Detoxification and export of xenobiotics are crucial for the maintenance of cellular homeostasis and protection against carcinogenic agents (6). Nrf2, a member of the cap 'n' collar family of basic region leucine zipper transcription factors (7), is a key transcriptional regulator of detoxification enzymes, transporters, and antioxidative molecules. Nrf2 forms heterodimers with small Maf proteins (MafF, MafG, and MafK), binds to antioxidant response elements (AREs),^2^ and activates transcription of target genes. Mice lacking Nrf2 fail to induce phase II detoxifying enzymes and antioxidative molecules in response to oxidative stress, indicating that Nrf2 has a critical role in cellular defense against xenobiotics and oxidative stress (8).

ARE-mediated transcriptional activities are regulated by the combinations and relative levels of cap 'n' collar molecules and small Maf proteins. The Nrf2-small Maf heterodimer is essential for the activation of ARE-mediated transcription. On the other hand, small Maf homodimers have been reported to suppress ARE-mediated transcription (7). Other cap 'n' collar molecules, including Bach1, form heterodimers with small Maf proteins and suppress ARE-mediated transcription (9). For example, the MafK-Bach1 heterodimer interacts with AREs in the enhancer region of the heme oxygenase-1 (*HO-1*) gene and suppresses its transcription. Heme, an inducer of *HO-1*, displaces Bach1 from the AREs, which is followed by binding of Nrf2 to the AREs, and increases in *HO-1* expression (10–12). HO-1 catalyzes the rate-limiting step in heme catabolism and generates carbon monoxide, ferric iron, and biliverdin. Carbon monoxide and ferric iron can activate Nrf2, suggesting that HO-1 acts as a cytoprotective factor in both suppression of oxidative stress and activation of Nrf2 (13–15).

In this study, we examined the effect of TGF-β on the expression of *HO-1*. We found that TGF-β induces expression of *MafK* and *Bach1* and that these genes are essential for suppression of *HO-1* by TGF-β signaling.

## EXPERIMENTAL PROCEDURES

### 

#### 

##### Cells and Culture

293T and NMuMG cells were obtained from the American Type Culture Collection. These cells were cultured in Dulbecco's modified Eagle's medium (Invitrogen) supplemented with 10% fetal bovine serum (FBS) and 1% penicillin-streptomycin solution (Invitrogen). Mouse mammary carcinoma JygMC(A) cells were cultured as described previously (16).

##### DNA Constructs

Expression constructs encoding ALK5T204D and FLAG-Smads (17, 18) and the luciferase reporters pNQO1-ARE-luc (19) and pHO1-luc (20) were described previously. cDNAs for Nrf2, MafF, MafG, MafK, and Bach1 were cloned into the pcDEF3 vector before use. For establishment of cells stably expressing FLAG-MafK or FLAG-Bach1, corresponding cDNAs were cloned into the pCAGIP vector (21).

##### DNA Transfection

293T and NMuMG cells were transfected using FuGENE 6 transfection reagent (Roche Diagnostics) or Lipofectamine 2000 (Invitrogen) following the manufacturers' recommendations. For establishment of cell lines stably expressing MafK or Bach1, NMuMG cells were transfected with pCAGIP-FLAG-MafK or pCAGIP-FLAG-Bach1, respectively; cloned; and maintained in the presence of puromycin (1 μg/ml; Sigma).

##### RNA Interference

NMuMG and JygMC(A) cells were transfected with 40 nm small interfering RNA (siRNA) directed against MafK or Bach1 using Lipofectamine 2000 (Invitrogen). In the case of double knockdown of MafK and Bach1, cells were transfected with a 40 nm concentration of each siRNA. Sequences of siRNA are listed in Table 1. Control siRNA was purchased from Invitrogen (Stealth^TM^ RNAi Negative Universal Control Medium). A pSUPER-puro vector (Oligoengine) expressing a short hairpin RNA against human and mouse Smad4 (pSUPER-sh-Smad4) was described previously (22). NMuMG cells transfected with pSUPER-sh-Smad4 were cloned and maintained in the presence of puromycin (1 μg/ml).

**TABLE 1:** **Sequence information for interference RNA**

Name	Sequence
**siRNA MafK 1**	
Sense	5′-CGAUGAUGAGCUGGUGUCCAUGUCA-3′
Antisense	5′-UGACAUGGACACCAGCUCAUCAUCG-3′

**siRNA MafK 2**	
Sense	5′-GGGCUAAUGUCUGUGUUCCUGUGUG-3′
Antisense	5′-CACACAGGAACACAGACAUUAGCCC-3′

**siRNA Bach1 1**	
Sense	5′-GAAGGCUGCUCAAGCAACUUGGAAA-3′
Antisense	5′-UUUCCAAGUUGCUUGAGCAGCCUUC-3′

**siRNA Bach1 2**	
Sense	5′-ACUGUGAGGUGAAGCUGCCAUUCAA-3′
Antisense	5′-UUGAAUGGCAGCUUCACCUCACAGU-3′

##### Luciferase Assay

Luciferase activities were measured using Luciferase Assay Systems (Promega) using a luminometer (MicroLumat, Berthold). The obtained luciferase activities were normalized to β-galactosidase activities of cotransfected pcDNA1.2/V5-GW/*lacZ* (Invitrogen).

##### Reverse Transcription and Polymerase Chain Reaction (RT-PCR)

Total RNA was prepared using ISOGEN (Nippon Gene). RT was performed using High Capacity RNA-to-cDNA Master Mix (Applied Biosystems), and PCR was performed using Ex Taq polymerase (Takara Bio). PCR primers are listed in Table 2.

**TABLE 2:** **Primers for semiquantitative PCR** GCSH, heavy chain of γ-glutamylcysteine synthetase.

Name	Sequence	Product length	Annealing temperature	Cycle number
		*bp*	°*C*	
**NQO1**				
Sense	5′-AGAAGAGAGGATGGGAGGTA-3′	354	55	25
Antisense	5′-TGGTGATAGAAAGCAAGGTC-3′			

**HO**-1				
Sense	5′-GGGTGACAGAAGAGGCTAAG-3′	319	55	22
Antisense	5′-CATTGGACAGAGTTCACAGC-3′			

**GCSH**				
Sense	5′-ATTGTTATGGCTTTGAGTGC-3′	353	55	25
Antisense	5′-GCATCATCCAGGTGTATTAA-3′			

**β-Actin**				
Sense	5′-GCTCATAGCTCTTCTCCAGGG-3′	396	55	22
Antisense	5′-TGAACCCTAAGGCCAACCGTG-3′			

**MafF**				
Sense	5′-AACACGCCGCACCTGTCGGA-3′	194	55	25
Antisense	5′-GACTTCTGCTTCTGCAGCTC-3′			

**MafG**				
Sense	5′-AGAAGGAGGAGCTGGAGAAG-3′	257	55	25
Antisense	5′-GCATCCGTCTTGGACTTTAC-3′			

**MafK**				
Sense	5′-AGCGATGATGAGCTGGTGTC-3′	206	55	25
Antisense	5′-AGCTTCTCCACCTCCTGCTG-3′			

**Bach1**				
Sense	5′-GTGCAGAGTAAAACCGTGAA-3′	499	55	25
Antisense	5′-AGTCATCTCCCAGGCTAATC-3′			

**Smad7**				
Sense	5′-GGAAGTCAAGAGGCTGTGTT-3′	296	55	25
Antisense	5′-GTCTTCTCCTCCCAGTATGC-3′			

##### Immunoprecipitation and Immunoblot Analysis

Cells were solubilized in lysis buffer (20 mm Tris-HCl, pH 7.5, 150 mm NaCl, 1% Nonidet P-40, 2000 KIU/ml aprotinin, 1 μg/ml leupeptin). After clearing by centrifugation, total cell lysates or immunoprecipitates obtained using the indicated antibodies were subjected to sodium dodecyl sulfate-polyacrylamide gel electrophoresis (SDS-PAGE). Proteins were electrotransferred to a mixed ester nitrocellulose membrane (Hybond-C Extra, GE Healthcare) and subjected to immunoblot analysis. Anti-Nrf2 (H-300, Santa Cruz Biotechnology), anti-HO-1 (Stressgen), anti-NAD(P)H:quinone oxidoreductase 1 (NQO1) (Abcam), anti-α-tubulin (Cell Signaling Technology), anti-β-actin (Sigma), anti-hemagglutinin (HA) (3F10, Roche Applied Science), anti-FLAG (M2, Sigma), anti-Smad2/3 (clone 18, BD Transduction Laboratories), anti-Smad4 (Santa Cruz Biotechnology), anti-phospho-Smad2 (23), anti-MafK (24), and anti-Bach1 (11) were used as primary antibodies for the immunoblot analysis. Reacted primary antibodies were detected with horseradish peroxidase-conjugated anti-mouse IgG or anti-rabbit IgG antibodies (GE Healthcare) and SuperSignal West Dura Extended Duration Substrate (Thermo Scientific). For reblotting, membranes were stripped according to the manufacturer's protocol.

##### Immunofluorescence Microscopy

To detect Nrf2 and Smad2, NMuMG cells were fixed in 4% formaldehyde. The fixed cells were permeabilized, and nonspecific protein binding was blocked by 1% bovine serum albumin (BSA) and 0.3% Triton X-100 in phosphate-buffered saline (PBS). Cells were incubated with a mixture of anti-Smad2 (BD Transduction Laboratories) and anti-Nrf2 (25) antibodies and then with Texas Red-conjugated anti-mouse IgG (Molecular Probes/Invitrogen) followed by Alexa Fluor 488-conjugated anti-rat IgG (Molecular Probes/Invitrogen). The nuclei were counterstained with 4′,6-diamidino-2-phenylindole (DAPI).

##### Cell Fractionation

Cells were washed with PBS and treated with ice-cold hypotonic buffer (20% glycerol, 20 mm HEPES, pH 7.6, 10 mm NaCl, 1.5 mm MgCl_2_, 0.2 mm EDTA, 0.1% Triton X-100, 25 mm β-glycerophosphate, 1 mm phenylmethylsulfonyl fluoride, 1 mm dithiothreitol, 20,000 KIU/ml aprotinin). Nuclei (pellet) and cytosol (supernatant) were separated by centrifugation (16,000 × *g*, 5 min). Nuclei were resuspended in SDS-PAGE sample buffer. Proteins in cytosolic fractions were precipitated with methanol and resuspended in SDS-PAGE sample buffer.

##### DNA Affinity Precipitation

Cells were lysed in 1% Nonidet P-40, 150 mm NaCl, 20 mm Tris-HCl, pH 7.5, 2000 KIU/ml aprotinin, 1 μg/ml leupeptin. Equal amounts of lysate protein were incubated with biotinylated double-stranded DNA oligonucleotides and poly(dI-dC) (Roche Applied Science) at 4 °C for 1 h. DNA-protein complexes were captured with streptavidin-agarose for 1 h and subjected to immunoblotting. The sequences of HO-1-ARE probe were as follows: sense, 5′-Bio-TTCGCTGAGTCATGGTTCCC-3′; antisense, 5′-GGGAACCATGACTCAGCGAA-3′ (20).

##### Chromatin Immunoprecipitation (ChIP)

ChIP was performed as described previously (17) with modifications. Cells were treated with TGF-β (5 ng/ml) for 1 h before treatment with *tert*-butylhydroquinone (*t*BHQ; 25 μm). After cross-linking with 1% formaldehyde at 37 °C for 15 min, cells were suspended in 500 μl of nuclear lysis buffer (1% SDS, 50 mm Tris-HCl, pH 8.1, 10 mm EDTA, 20,000 KIU/ml aprotinin, 1 μg/ml leupeptin) and sonicated. Soluble chromatin was diluted with 9 volumes of dilution buffer for immunoprecipitation (16.7 mm Tris-HCl, pH 8.1, 1.2 mm EDTA, 167 mm NaCl, 0.01% SDS, 1.1% Triton X-100, 20,000 KIU/ml aprotinin, 1 μg/ml leupeptin) and incubated with normal rabbit IgG, anti-Nrf2 (H-300, Santa Cruz Biotechnology), anti-MafK (24), anti-Bach1 (11), or anti-Smad2/3 (clone 18, BD Transduction Laboratories) antibody with end-over-end rotation at 4 °C overnight followed by incubation with 25 μl of Dynabeads^®^ Protein A (Invitrogen) at 4 °C for 1 h. DNA was extracted from the Dynabeads by means of phenol-chloroform extraction. PCR was performed using Ex Taq polymerase. The PCR primers are described in Table 3. Otherwise, quantitative PCR was performed using qPCR MasterMix for SYBER Green I (Applied Biosystems) and the ABI7500 Fast Sequence Detection system. All samples were run in triplicate in each experiment. Primer sequences are listed in Table 4.

**TABLE 3:** **Primers for ChIP analysis**

Name	Sequence	Product length	Annealing temperature	Cycle number
		*bp*	°*C*	
***HO-1*** promoter (E1)				
Sense	5′-TGAAGTTAAAGCCGTTCCGG-3′	183	53	35
Antisense	5′-AGCGGCTGGAATGCTGAGT-3′			

***HO-1*** promoter (E2)				
Sense	5′-GGGCTAGCATGCGAAGTGAG-3′	201	53	35
Antisense	5′-AGACTCCGCCCTAAGGGTTC-3′			

**TABLE 4:** **Primers for ChIP analysis and quantitative PCR**

Name	Sequence	Product length
		*bp*
***HO-1*** promoter (E2)		
Sense	5′-GGGCAGTCTTAAGCAATCCA-3′	146
Antisense	5′-AAGGGTTCAGTCTGGAGCAA-3′	

**Smad7**		
Sense	5′-TAGAAACCCGATCTGTTGTTTGCG-3′	132
Antisense	5′-CCTCTGCTCGGCTGGTTCCACTGC-3′	

## RESULTS

### 

#### 

##### TGF-β Suppresses Electrophile-inducible Expression of HO-1

**FIGURE 1.**
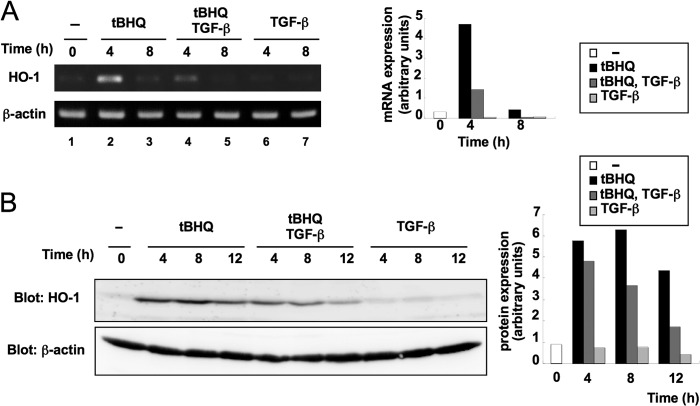
**TGF-β suppresses *t*BHQ-inducible expression of the *HO-1*.** *A*, TGF-β suppresses *t*BHQ-inducible mRNA expression of *HO-1*. NMuMG cells were treated with TGF-β (5 ng/ml) for 1 h before stimulation with *t*BHQ (25 μm) and incubated for the indicated times. HO-1 and β-actin mRNAs were detected by semiquantitative RT-PCR (*left panel*). Representative mRNA expression was quantified using NIH ImageJ and normalized to β-actin (*right panel*). *B*, NMuMG cells were treated as in *A*. Immunoblot analysis was performed using anti-HO-1 antibody. β-Actin was examined as a loading control (*left panel*). Quantification of the protein levels was performed using NIH ImageJ and normalized to β-actin (*right panel*). All experiments were repeated more than three times to confirm their reproducibility.

To examine the effect of TGF-β on expression of *HO-1*, NMuMG cells were treated with *t*BHQ in the absence or presence of TGF-β signaling. mRNA levels of *HO-1* were highly increased 4 h after *t*BHQ stimulation (Fig. 1*A*, *lanes 2* and *3*). However, pretreatment of the cells with TGF-β significantly reduced the induction (Fig. 1*A*, *lanes 4* and *5*). TGF-β alone had no detectable effect on the basal expression levels of *HO-1* (Fig. 1*A*, *lanes 6* and *7*). A representative result of the densitometric quantification of normalized mRNA levels is shown in Fig. 1*A*, *right panel*. TGF-β-mediated suppression of *HO-1* was also examined at the protein level (Fig. 1*B*). These results indicate that TGF-β suppresses *t*BHQ-inducible expression of *HO-1* in NMuMG cells.

##### TGF-β Does Not Affect the Stabilization or Nuclear Accumulation of Nrf2

**FIGURE 2.**
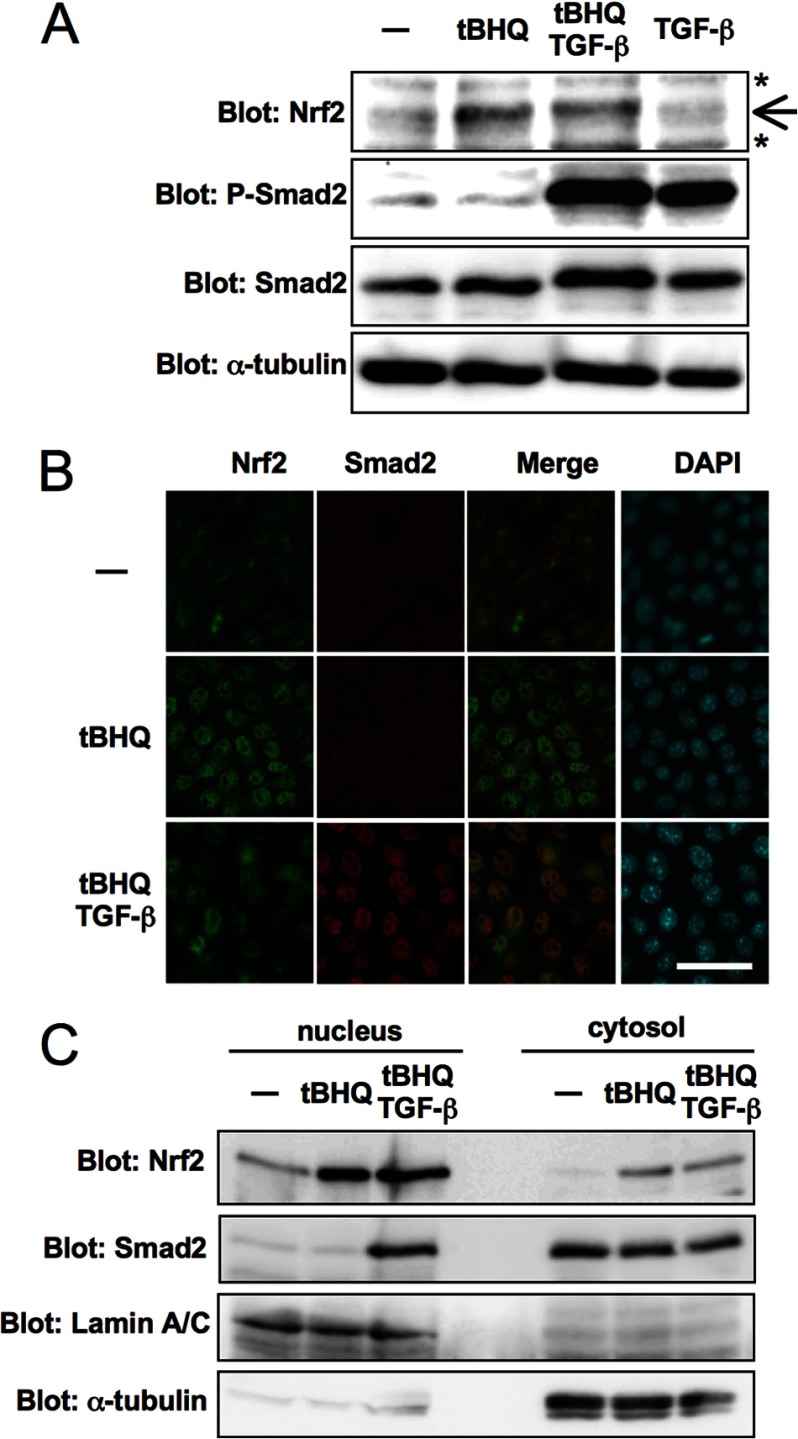
**TGF-β does not affect the stabilization and nuclear accumulation of Nrf2.** *A*, NMuMG cells were treated with TGF-β (5 ng/ml) for 1 h before stimulation with *t*BHQ (25 μm) and incubated for an additional 4 h. Immunoblot analysis was performed using anti-Nrf2, -phospho-Smad2 (*P-Smad2*), -Smad2 and -α-tubulin antibodies as indicated. *Arrow*, specific band for Nrf2; *, nonspecific bands. *B*, NMuMG cells were treated as in *A*. After fixation, cells were serially stained with anti-Nrf2 (*green*) and anti-Smad2 (*red*) antibodies. Nuclei were counterstained with DAPI. *Scale bar*, 50 μm. *C*, NMuMG cells were treated as in *A*. Nuclear and cytosolic fractions were isolated and analyzed by immunoblotting using antibodies for Nrf2 and Smad2. Lamin A/C was used as a nuclear protein marker, and α-tubulin was used as a cytosolic protein marker.

To examine how TGF-β affects *HO-1* expression, we examined the expression and subcellular localization of Nrf2 proteins in NMuMG cells. Because Nrf2 activates the transcription of *HO-1*, we assumed that TGF-β would affect the accumulation or nuclear translocation of Nrf2. However, as shown in Fig. 2, neither the stabilization (Fig. 2*A*) nor the nuclear localization of Nrf2 (Fig. 2, *B* and *C*) was affected by TGF-β.

##### TGF-β Induces MafK and Bach1

**FIGURE 3.**
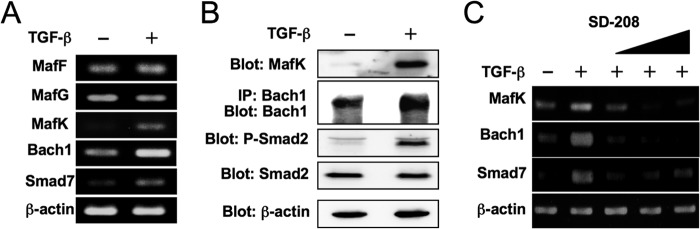
**TGF-β induces MafK and Bach1.** *A*, NMuMG cells were treated with TGF-β (5 ng/ml) for 1 h. mRNA for the small Maf family of transcription factors (MafF, MafG, and MafK), Bach1, Smad7, and β-actin was detected by semiquantitative RT-PCR. *B*, NMuMG cells were treated with TGF-β (5 ng/ml) for 4 h. Immunoblot analysis was performed using anti-MafK, -Bach1, -phospho-Smad2 (*P-Smad2*), -Smad2, and -β-actin antibodies as indicated. Bach1 was detected after immunoprecipitation (*IP*) with anti-Bach1 antibody to increase the sensitivity. *C*, NMuMG cells were treated with a TGF-β type I receptor kinase inhibitor, SD-208 (0, 0.03, 0.1, and 0.3 μm) for 30 min before treatment with TGF-β (5 ng/ml) for 1 h. MafK, Bach1, Smad7, and β-actin mRNAs were detected by semiquantitative RT-RCR.

Nrf2 activates transcription of target genes by binding to AREs together with small Maf proteins. However, if small Maf levels rise exceedingly, small Maf homodimers can compete with Nrf2-small Maf heterodimers for the binding to AREs (7). Otherwise, heterodimers of small Mafs and other cap 'n' collar-type transcriptional regulators such as Bach1 can compete for the binding (9). Because TGF-β did not affect the nuclear accumulation of Nrf2, we next examined the effect of TGF-β on the expression of small Mafs and Bach1 and found that TGF-β increased MafK and Bach1 at both mRNA and protein levels (Fig. 3, *A* and *B*). Induction of MafK and Bach1 was suppressed by a TGF-β type I receptor kinase inhibitor, SD-208 (Fig. 3*C*).

##### MafK and Bach1 Regulate Induction of HO-1

**FIGURE 4.**
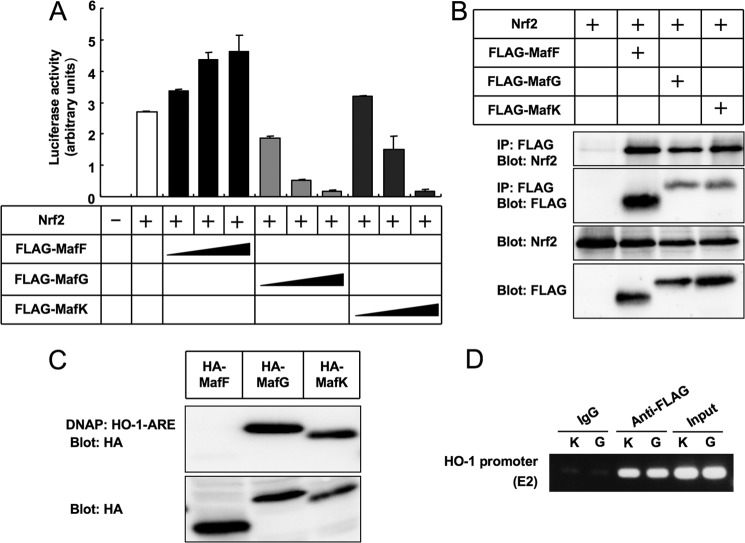
**MafK and MafG, but not MafF, suppress transcriptional activity of *HO-1*.** *A*, pHO1-luc reporter activities were activated by overexpression of Nrf2, and the effects of MafF, MafG, and MafK were examined. *Error bars* represent S.D. *B*, interaction between Nrf2 and small Mafs was examined by coprecipitation assays in 293T cells. MafF, MafG, and MafK all coprecipitated Nrf2. *C*, binding of small Mafs to AREs from *HO-1*. HA-tagged MafF, MafG, and MafK were expressed in 293T cells, and the cell lysates were incubated with biotinylated double-stranded DNA fragments (Table 3) and precipitated with avidin beads. Coprecipitated proteins were detected with anti-HA antibody. *D*, chromatin immunoprecipitation analysis using anti-FLAG antibody detected binding of MafK and MafG to the *HO-1* promoter region including ARE (E2) in NMuMG-MafK (*K*) and NMuMG-MafG (*G*) cells, respectively. *DNAP*, DNA affinity precipitation; *IP*, immunoprecipitation.

**FIGURE 5.**
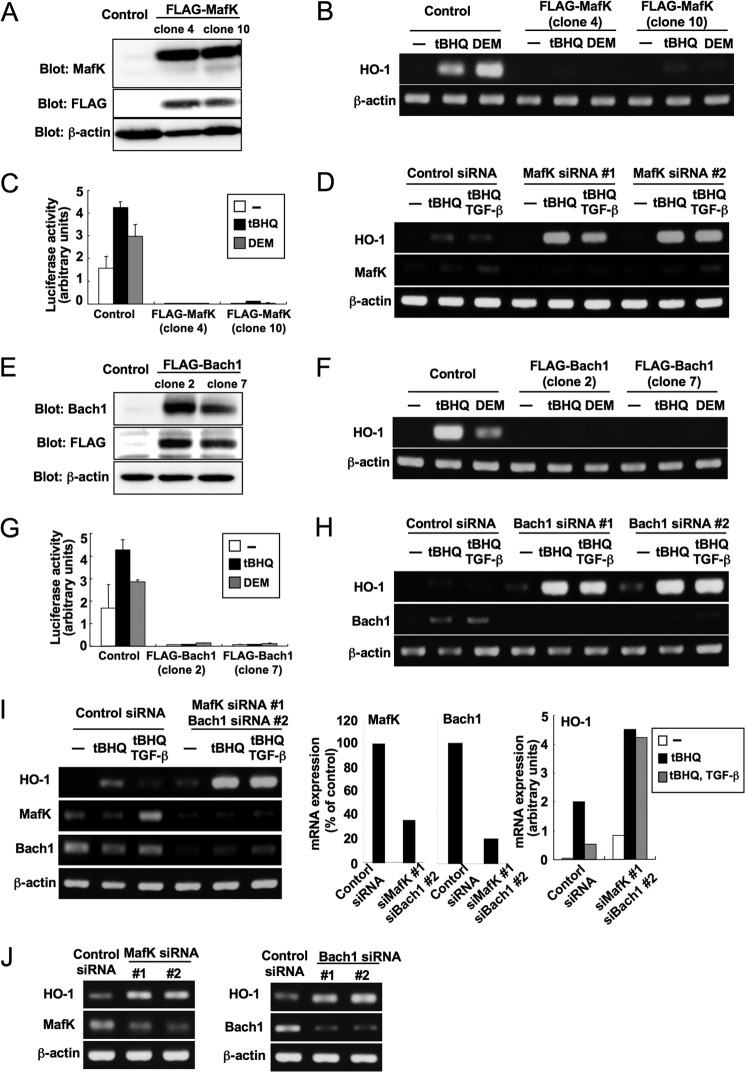
**MafK and Bach1 regulate expression of *HO-1*.** *A* and *B*, MafK suppresses *HO-1* induction by *t*BHQ or DEM. *A*, establishment of NMuMG cells stably expressing FLAG-MafK (clones 4 and 10). *Control* represents NMuMG cells transfected with empty vector. *B*, impaired induction of *HO-1* in NMuMG-MafK cells. NMuMG-MafK cells were treated with *t*BHQ (25 μm) or DEM (100 μm) for 4 h. HO-1 and β-actin mRNAs were detected by semiquantitative RT-PCR. *C*, pHO-1-luc activities are inactivated in NMuMG-MafK cells. Six hours after transfection with pHO-1-luc, cells were treated with *t*BHQ (25 μm) or DEM (100 μm) for 12 h. *Error bars* represent mean ± S.D. *D*, knockdown of MafK enhances induction of *HO-1*. NMuMG cells were transfected with MafK siRNA 1 or 2 as described under “Experimental Procedures.” Cells were then treated with TGF-β (5 ng/ml) for 1 h and stimulated with *t*BHQ (25 μm) for 4 h. HO-1, MafK, and β-actin mRNAs were detected by semiquantitative RT-PCR. *E* and *F*, Bach1 suppresses induction of *HO-1* by *t*BHQ and DEM. *E*, establishment of NMuMG cells stably expressing FLAG-Bach1 (clones 2 and 7). *F*, impaired induction of *HO-1* in NMuMG-Bach1 cells. NMuMG-Bach1 cells were treated with *t*BHQ (25 μm) or DEM (100 μm) for 4 h. HO-1 and β-actin mRNAs were examined by semiquantitative RT-PCR. *G*, pHO-1-luc activities are suppressed in NMuMG-Bach1 cells. Six hours after transfection with pHO-1-luc, cells were treated with *t*BHQ (25 μm) or DEM (100 μm) for 12 h. *Error bars* represent mean ± S.D. *H*, knockdown of Bach1 enhances induction of *HO-1* and impairs the suppressive effects of TGF-β. NMuMG cells were transfected with Bach1 siRNA 1 or 2 as described under “Experimental Procedures.” Cells were treated with TGF-β (5 ng/ml) for 1 h before treatment with *t*BHQ (25 μm) for 4 h. HO-1, Bach1, and β-actin mRNAs were detected by semiquantitative RT-PCR. *I*, double knockdown of MafK and Bach1 enhances induction of *HO-1* and almost completely impairs the suppressive effects of TGF-β. NMuMG cells were transfected with MafK siRNA 1 and Bach1 siRNA 2 as described under “Experimental Procedures.” Cells were then treated with TGF-β (5 ng/ml) for 1 h before treatment with *t*BHQ (25 μm) for 4 h. HO-1, MafK, Bach1, and β-actin mRNAs were detected by semiquantitative RT-PCR. Representative mRNA expression was quantified using NIH ImageJ and normalized to β-actin (*right panel*). *J*, knockdown of MafK or Bach1 enhances expression of *HO-1* in breast cancer cells. JygMC(A) cells were transfected with MafK siRNA or Bach1 siRNA as indicated. HO-1, MafK, Bach1, and β-actin mRNAs were detected by semiquantitative RT-RCR.

We next examined the effect of MafF, MafG, and MafK on pHO1-luc activities. Transiently expressed MafG and MafK, but not MafF, suppressed the reporter activities (Fig. 4*A*). MafF, MafG, and MafK all formed heterodimers with Nrf2 (Fig. 4*B*), but when small Maf proteins were independently expressed, MafK and MafG, but not MafF bound to *HO-1* ARE DNA fragments (Fig. 4*C*). We then established NMuMG cell lines with stable expression of MafK, MafG, or Bach1. Binding of MafK and MafG to *HO-1* ARE (E2) was confirmed by chromatin immunoprecipitation (Fig. 4*D*). In functional analyses, both *t*BHQ and diethyl maleate (DEM) induced *HO-1* expression in NMuMG cells. However, these effects were completely blocked by overexpression of MafK (Fig. 5, *A* and *B*). MafK also decreased pHO1-luc activities in both the absence and the presence of *t*BHQ or DEM (Fig. 5*C*). On the other hand, reduction of MafK by siRNA strikingly enhanced *t*BHQ-inducible expression of *HO-1* mRNA (Fig. 5*D*). However, stable overexpression of MafG did not suppress *t*BHQ- and DEM-inducible expression of *HO-1* (supplemental Fig. 2). We also established NMuMG cells stably expressing FLAG-Bach1 (Fig. 5*E*). Bach1 had a nearly identical effect as that of MafK on *HO-1* expression (Fig. 5, *E–H*) except for a stronger induction of *HO-1* in the absence of both electrophiles and TGF-β (Fig. 5*H*). Furthermore, double knockdown of MafK and Bach1 highly increased both the basal and the *t*BHQ-inducible expression of *HO-1* and abolished the suppressive effect of TGF-β on *t*BHQ-inducible *HO-1* expression (Fig. 5*I*). Endogenous MafK and Bach1 also suppressed *HO-1* in breast cancer cells. Knockdown of MafK or Bach1 in JygMC(A) cells clearly increased expression of *HO-1* (Fig. 5*J*).

##### Recruitment of Nrf2, MafK, and Bach1 to ARE Sites in the HO-1 Promoter

**FIGURE 6.**
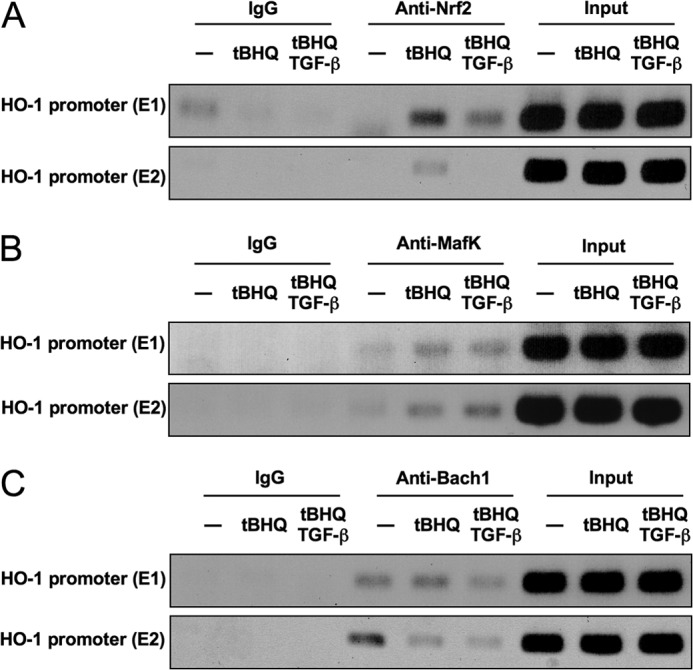
**Effects of TGF-β on the recruitment of Nrf2, MafK, and Bach1 to AREs (E1 and E2) in the *HO-1* promoter.** NMuMG cells were treated with TGF-β (5 ng/ml) for 1 h before stimulation with *t*BHQ (25 μm) for 4 h. After fixation, soluble chromatin was immunoprecipitated using anti-Nrf2 (*A*), anti-MafK (*B*), or anti-Bach1 (*C*) antibody as indicated. *HO-1* promoter fragments containing AREs (E1 and E2) were amplified by PCR. *Input*, total chromatin solution analyzed as a control.

ARE sites (E1 and E2) in the promoter region of *HO-1* are essential for its transcriptional regulation. To determine whether changes in *HO-1* expression are associated with altered binding of Nrf2, MafK, and Bach1 to ARE sites *in vivo*, we performed chromatin immunoprecipitation assays. When cells were treated with *t*BHQ, Nrf2 binding to both E1 and E2 (mainly to E1) increased. These interactions were reduced when cells were treated with TGF-β before *t*BHQ stimulation (Fig. 6*A*). Binding of MafK to E1 and E2 increased after *t*BHQ stimulation together with Nrf2, but its binding to E2 was not decreased by TGF-β treatment (Fig. 6*B*). In contrast, Bach1 binding to E2 was highest in the absence of *t*BHQ and was reduced when cells were treated with *t*BHQ (Fig. 6*C*).

##### Binding of Smad3 and MafK on ARE Sites in the HO-1 Promoter

**FIGURE 7.**
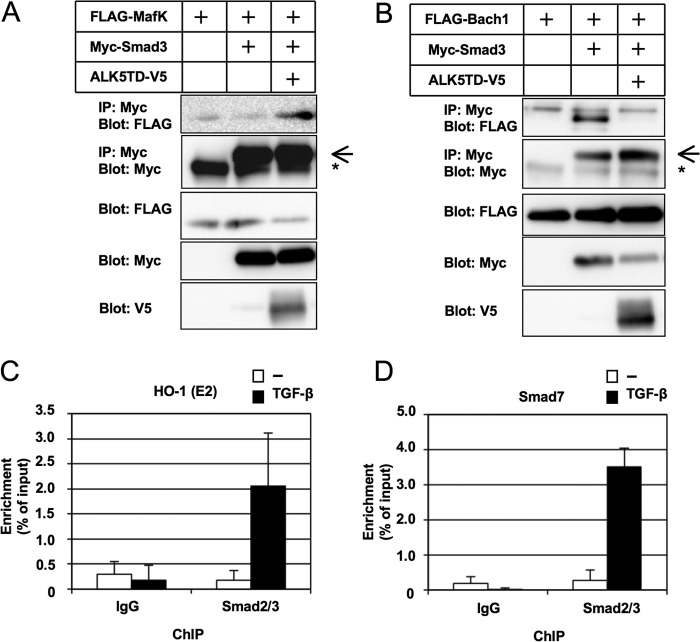
**Cooperative functions of TGF-β/Smad signaling with MafK and Bach1.** *A* and *B*, binding of Smad3 to MafK (*A*) and Bach1 (*B*). FLAG-MafK, FLAG-Bach1, Myc-Smad3, and a constitutively active TGF-β type I receptor (ALK5TD-V5) were transfected to 293T cells as indicated, and binding of MafK or Bach1 and Smad3 was examined by immunoprecipitation (*IP*) with anti-Myc antibody followed by immunoblotting with anti-FLAG antibody. *Arrow*, Myc-Smad3; *, IgG. *C* and *D*, chromatin immunoprecipitation analyses using anti-Smad2/3 antibody detected binding of Smad2/3 to the *HO-1* promoter region including ARE (E2) in NMuMG cells (*C*). Binding of Smad2/3 to the Smad7 promoter region was used as a positive control (*D*). *Error bars* represent S.D.

Binding of MafK or Bach1 and Smad3 was examined by coprecipitation assays. Both MafK and Bach1 bound to Smad3. MafK bound to Smad3 in the presence of TGF-β signaling. On the other hand, Bach1 bound to Smad3 in the absence of TGF-β signaling (Fig. 7, *A* and *B*). Binding of Smad2/3 on ARE (E2) in the promoter region of *HO-1* was detected by chromatin immunoprecipitation assays in the presence of TGF-β signaling (Fig. 7*C*).

## DISCUSSION

In this study, we proved that TGF-β suppresses the electrophile-inducible transcriptional activation of *HO-1*. This is the first report on the regulation of the Nrf2-mediated oxidative stress responses by TGF-β signaling. Intriguingly, TGF-β did not reduce the nuclear accumulation of Nrf2 provoked by electrophiles, but the recruitment of Nrf2 to AREs (E1 and E2) in the promoter region of *HO-1* was clearly suppressed by TGF-β. Consistent with the previous report that MafK-Bach1 heterodimers repress *HO-1* expression (12), knockdown of MafK and Bach1 enhanced *HO-1* expression and abolished the suppressive effect by TGF-β (Fig. 5*I*). These results suggest that TGF-β signaling generally antagonizes cytoprotective responses mediated by the Keap1-Nrf2 system. Therefore, we further investigated the effect of TGF-β on Nrf2-mediated activation of other Nrf2 target genes. NQO1 and the heavy and light chains of γ-glutamylcysteine synthetase were examined, and all of these target genes were suppressed by TGF-β, although the extents of the suppression were different in different genes (data not shown). Furthermore, the constitutively active TGF-β type I receptor ALK5T204D significantly suppressed Nrf2-mediated activation of pNQO1-ARE-luc together with Smad3 (data not shown). The reporter activity was further suppressed by the addition of Smad4. These results suggested that TGF-β suppresses the transcriptional activity of Nrf2 through activation of the Smad signaling pathway. However, suppression of these Nrf2-target genes was not canceled by knockdown of MafK and Bach1 (data not shown). *HO-1* was the only gene in which suppression by TGF-β was canceled by knockdown of MafK and Bach1. Therefore, TGF-β probably suppresses different target genes in different molecular mechanisms. Consistent with this notion, both *HO-1* and *NQO1* are regulated by Nrf2, and these genes contain ARE sites in their promoter regions, but Bach1 interacts specifically with AREs in *HO-1*. Subtle differences in AREs or their flanking sequences affect the binding affinities of the Maf-containing dimers, resulting in different contributions of each dimer to the ARE-dependent gene regulation. Indeed, both Nrf2-MafG heterodimer and MafG homodimer bind to the consensus Maf recognition element with high affinity but bind differentially to the suboptimal binding sequences degenerated from the consensus (26, 27). Different Maf complexes may be differently regulated by TGF-β signaling.

A discrepancy between the nuclear accumulation and transcriptional activity of Nrf2 has been reported. When human aortic endothelial cells were exposed to oscillating flow, Nrf2 accumulated in the nuclei but did not activate stress response genes. In contrast, when the cells were exposed to laminar flow, Nrf2 accumulated in the nuclei and activated its target genes (28). The analogous finding of the current study suggests that levels of small Mafs and Bach1 or other related transcriptional factors might be involved in nuclear regulation of Nrf2 activities.

The functional analyses described above clearly indicated that MafK and Bach1 are essential for the suppression of *HO-1* by TGF-β signaling (Fig. 5). ChIP analyses (Fig. 6) also revealed that *t*BHQ treatment substitutes Nrf2 for Bach1 in the E1 and E2 elements of *HO-1* and that TGF-β reduces Nrf2 in both elements. However, TGF-β signaling increased MafK binding only marginally, and Bach1 binding to both E1 and E2 was reduced. These results suggest that the displacement of Nrf2 from E1 and E2 was not simply the result of the direct competitive binding between Nrf2 and MafK/Bach1. Consistent with this, we detected Smad2/3 on ARE (E2) in the presence of TGF-β signaling (Fig. 7). The exact molecular mechanism of MafK, Bach1, Smads, and possibly MafG in the TGF-β-dependent suppression of *HO-1* remains to be elucidated.

TGF-β markedly elevated expression of MafK and Bach1 in NMuMG cells. Transcriptional regulation of tissue-specific expression of the *MafK* gene was analyzed previously in transgenic mice harboring the *lacZ* gene as a reporter. Two alternative promoters were identified in the *MafK* gene, and the upstream and downstream promoters mediate the mesodermal and neuronal expressions, respectively (29, 30). A hematopoietic enhancer was also identified in the 3′-region of the *MafK* gene (31). However, the regulatory regions responsible for the induction by TGF-β have not been identified. We found that Smad4 was indispensable for the expression of MafK and suppression of *HO-1* by TGF-β (supplemental Fig. 1, C and E). Conversely, Bach1 expression was constitutively activated by knockdown of Smad4 (supplemental Fig. 1E).

The regulatory mechanisms of Bach1 function identified so far are mainly at the posttranscriptional level, *i.e.* changes in DNA binding affinity, subcellular localization, and protein stability (10, 32, 33). One report described the transcriptional regulation of *BACH1* examined in a reporter assay in K562 cells (34). A GC box residing in the promoter region was critical for the promoter activity, and Sp1 was a transactivating factor binding to the GC box, which could be a target of TGF-β signaling. Transcriptional regulation of *Bach1* by TGF-β signaling might constitute a novel layer of the regulation of Bach1 function *in vivo*.

TGF-β signaling is highly enhanced in many pathological conditions including precancerous lesions associated with ulcerative colitis and viral hepatitis (35, 36). The present study demonstrated that TGF-β markedly suppresses a cytoprotective gene, *HO-1*. It should be noted that a single nucleotide polymorphism in the enhancer region of *HO-1* that affects *HO-1* expression has been implicated in an increased incidence of lung cancer (37). These molecular epidemiological data suggest that suppression of *HO-1* may accelerate cancer initiation and progression. Therefore, TGF-β might promote cancer initiation in precancerous lesions or expedite cancer progression through suppression of Nrf2 target genes including *HO-1*.

## Supplementary Material

Supplemental Data

## Footnotes

2 The abbreviations used are:

ARE: antioxidant response element
ALK5: activin receptor-like kinase-5
DEM: diethyl maleate
HO-1: heme oxygenase-1
NQO1: NAD(P)H:quinone oxidoreductase 1
*t*BHQ: *tert*-butylhydroquinone
KIU: kallikrein-inactivating unit.
