# Supplementary material for: Transforming Growth Factor-β Induces Transcription Factors MafK and Bach1 to Suppress Expression of the Heme Oxygenase-1 Gene
Source: J Biol Chem. 2013 Jun 4;288(28):20658–67. doi: 10.1074/jbc.M113.450478 (PMC3711329; doi:10.1074/jbc.M113.450478)
Supplement: Supplemental Data [file supp_288_28_20658__index.html]

Transforming Growth Factor-β Induces transcription factors MafK and Bach1 to Suppress Expression of the Heme Oxygenase-1 Gene — Transforming Growth Factor-β Induces Transcription Factors MafK and Bach1 to Suppress Expression of the Heme Oxygenase-1 Gene — TGF-β Induces MafK and Bach1 to Suppress HO-1 — Supplemental Data 

# Transforming Growth Factor-β Induces Transcription Factors MafK and Bach1 to Suppress Expression of the Heme Oxygenase-1 Gene

## 

**Files in this Data Supplement:**

- Supplemental Figure Legends (.pdf, 73 KB) - Supplemental Figure Legends for reviewers and readers with special interest on this work.
- Supplemental Figures (.pdf, 2.6 MB) - Supplemental Figures for reviewers and readers with special interest on this work.
