# Supplementary material for: Transforming Growth Factor-β Induces Transcription Factors MafK and Bach1 to Suppress Expression of the Heme Oxygenase-1 Gene
Source: J Biol Chem. 2013 Jun 4;288(28):20658–67. doi: 10.1074/jbc.M113.450478 (PMC3711329; doi:10.1074/jbc.M113.450478)
Supplement: Supplemental Data [file supp_M113.450478_jbc.M113.450478-1.pdf]

LEGENDS FOR SUPPLEMENTARY FIGURES

SUPPLEMENTARY FIGURE 1. Smad4 is required for TGF-β-inducible suppression of Nrf2 activities. **A** Immunoblot analyses for Smad4, phospho-Smad2, Smad2/3, and β-actin in NMuMG cells transfected with pSUPER-sh-Smad4. Control represents NMuMG cells transfected with empty vector. **B** Functional confirmation of the Smad4-knockdown using impaired induction of Smad7, a known TGF-β/Smad-responsive gene, as a readout. NMuMG sh-Smad4 cells were treated with TGF-β (2.5 ng/ml) for the indicated times. Smad7 and β-actin mRNAs were detected by semiquantitative RT-PCR. **C** Impaired suppression of HO-1 mRNA in response to TGF-β. NMuMG sh-Smad4 cells were treated with TGF-β (5 ng/ml) for one hour before stimulation with *t*BHQ (25 μM) for 4 hours. HO-1 and β-actin mRNAs were detected by semiquantitative RT-PCR. **D** Impaired suppression of pHO1-luc activities in NMuMG sh-Smad4 cells. Twenty-four hours after transfection with pHO1-luc, cells were treated with TGF-β (5 ng/ml) for one hour before stimulation with *t*BHQ (25 μM) for 12 hours. Error bars represent mean ± SD. **E** NMuMG sh-Smad4 cells were treated with TGF-β (5 ng/ml) as indicated. Induction of MafK was impaired in NMuMG sh-Smad4 cells. On the other hand, constitutively high levels of expression of Bach1 was obtained in NMuMG sh-Smad4 cells.

SUPPLEMENTARY FIGURE 2. Constitutive overexpression of MafG does not suppress *HO-1* expression. NMuMG-MafG cells were treated with *t*BHQ (25 μM) or DEM (100 μM) for 4 hours. HO-1, MafG and β-actin mRNAs were detected by semiquantitative RT-PCR.
