# Supplementary figures and images for: Transforming Growth Factor-β Induces Transcription Factors MafK and Bach1 to Suppress Expression of the Heme Oxygenase-1 Gene
Source: J Biol Chem. 2013 Jun 4;288(28):20658–67. doi: 10.1074/jbc.M113.450478 (PMC3711329; doi:10.1074/jbc.M113.450478)

A

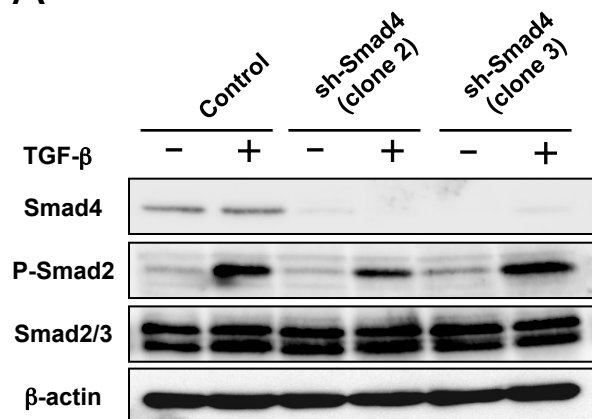

B

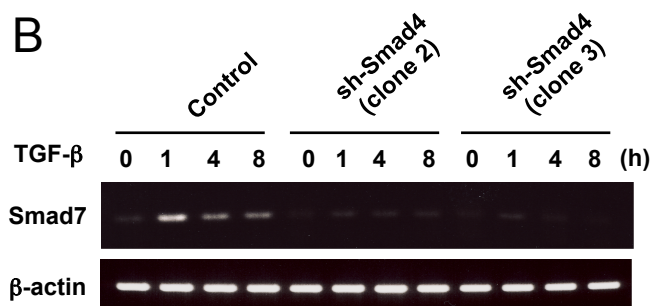

E

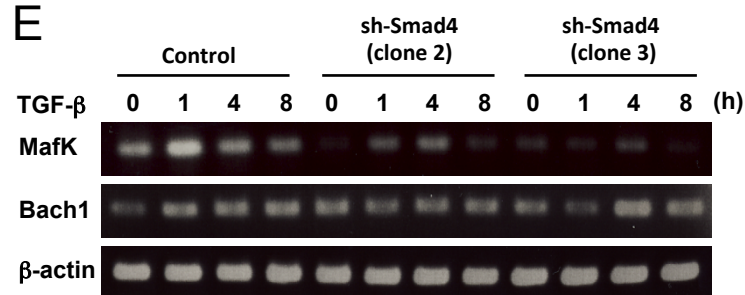

C

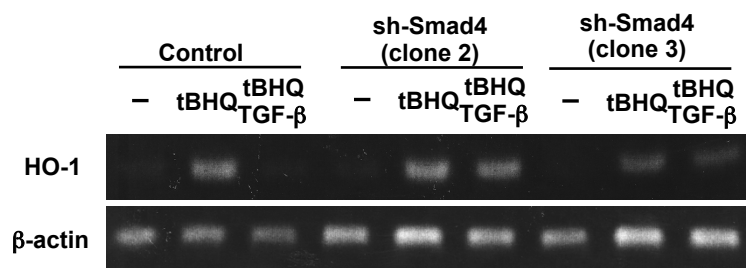

D

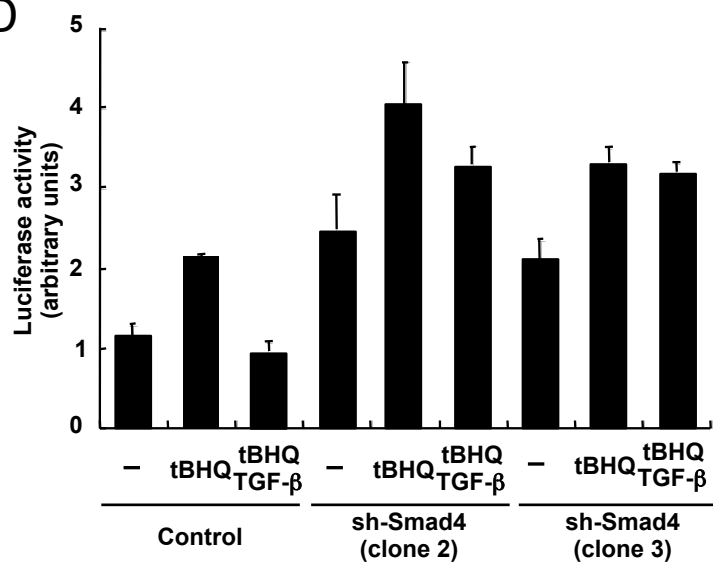

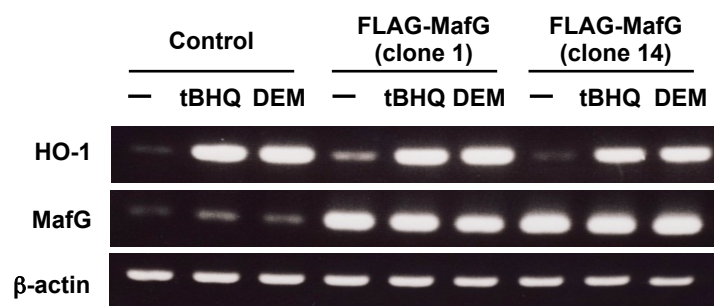

Supplementary  
Figure 2

Supplement: Supplemental Data [file supp_M113.450478_jbc.M113.450478-2.pdf]
